# Optimization of Multi-Component Cement Mortar Using a Taguchi Orthogonal Array Design

**DOI:** 10.3390/ma19153269

**Published:** 2026-08-02

**Authors:** Saruul Shinebayar, Yipei Chen, Jin Kim, Jung-Geun Han

**Affiliations:** 1 Department of Civil Engineering, Chung-Ang University, Seoul 06974, Republic of Korea; saruul202022@cau.ac.kr; 2 Department of Architecture and Building Science, Chung-Ang University, Seoul 06974, Republic of Korea; jinuibi@cau.ac.kr; 3 Department of Intelligent Energy Industry, Chung-Ang University, Seoul 06974, Republic of Korea; kimjin4311@cau.ac.kr

**Keywords:** multi-component cement mortar, taguchi orthogonal array design, supplementary cementitious materials, compressive strength

## Abstract

Developing low-carbon cement-based materials is a critical strategy for reducing the carbon footprint of the construction sector. This study optimized multi-component cement mortars incorporating natural zeolite (NZ), fly ash (FA), blast furnace slag (BFS), and calcium hydroxide (CH) using an L9 Taguchi orthogonal array and range analysis. Mechanical and physical properties, microstructural characteristics, statistical modeling and environmental impact were evaluated. The optimized mixture, OPT1 (10% NZ, 10% FA, 40% BFS, and 2% CH), achieved compressive strengths of 34.97 MPa and 62.67 MPa at 7 and 28 days, respectively, exceeding the control mortar by 8.4% at 28 days. The results indicated that supplementary cementitious materials (SCMs) reduced early-age strength due to the dilution effect; however, their pozzolanic and latent hydraulic reactions enhanced later-age strength development. OPT1 also demonstrated improved mechanical efficiency, reduced Global Warming Potential (GWP) by 35%, and achieved an eco-efficiency index (EEI) value of 124.6, which was 1.6 times that of the control mixture. These findings highlight the potential of the proposed system for high-performance and eco-efficient low-carbon cement mortars.

## 1. Introduction

The cement industry is one of the largest contributors to global greenhouse gas emissions due to the carbon-intensive nature of cement production. With rapid population growth and accelerating urbanization, global cement demand continues to increase, leading to a corresponding rise in CO_2_ emissions. Global CO_2_ emissions from cement production increased from approximately 0.7 billion tons in 2000 to 1.57 billion tons in 2023, accounting for nearly 8% of global total CO_2_ emissions [1,2,3]. Consequently, mitigating the environmental impact of cement-based construction materials has become a critical research priority, fueling the development of lower-carbon cement-based materials.

One of the most promising approaches for reducing the embodied carbon of cement-based composites is the partial replacement of Ordinary Portland Cement (OPC) with supplementary cementitious materials (SCMs), including natural pozzolans and industrial by-products [4,5,6,7]. SCMs not only reduce the amount of OPC required in cementitious binders, thereby lowering associated CO_2_ emissions, but can also improve the long-term mechanical performance and durability of cement-based materials. Natural pozzolans enhance later-age strength through pozzolanic reactions while lowering the environmental impact associated with cement production [8,9]. Similarly, industrial by-products such as fly ash (FA) and ground granulated blast furnace slag (BFS) are widely utilized to divert waste and improve material longevity while lowering embodied carbon emissions [10].

Among natural pozzolans, natural zeolite (NZ), particularly the clinoptilolite type, has attracted considerable attention because of its porous structure and high pozzolanic activity. During cement hydration, NZ reacts with calcium hydroxide (CH) to generate additional calcium silicate hydrate (C-S-H) and calcium aluminate hydrate (C-A-H) gels, thereby enhancing the microstructure and improving long-term strength development [11,12,13]. However, the relatively low CaO content of NZ limits its replacement level. Previous studies have reported that replacing 5–15% of cement with NZ generally improves compressive strength, whereas higher replacement levels often result in strength reduction. In addition, the porous structure of NZ increases water demand and tends to reduce workability, frequently requiring higher dosages of superplasticizer [11,13,14,15,16,17].

Fly ash can partially compensate for these limitations. Owing to its spherical particle shape and smooth surface texture, FA improves workability by reducing internal friction within fresh mixtures. Furthermore, its high silica (SiO_2_) and alumina (Al_2_O_3_) contents promote pozzolanic reactions with CH, resulting in a denser microstructure and enhanced long-term strength. Nevertheless, Class F fly ash generally contains less than 10% CaO, resulting in relatively low early-age reactivity. The addition of external calcium sources, such as CH, can promote the pozzolanic reaction of FA, accelerate hydration, and compensate for the slow strength development commonly observed during the first 28 days [18,19,20,21].

Ground granulated blast furnace slag provides additional advantages because of its latent hydraulic properties arising from its relatively high CaO content. Unlike NZ and FA, BFS exhibits both hydraulic and pozzolanic reactivity, allowing it to contribute to both early and later-age strength development. It also refines the pore structure, reduces permeability, and enhances resistance to aggressive chemical environments. Owing to its high reactivity, BFS can be incorporated at relatively high replacement levels, making it an effective constituent for reducing OPC content in mortars while maintaining mechanical performance [22,23,24,25].

Despite these advantages, high-volume replacement with SCMs also presents several challenges. The relatively slow hydration kinetics of FA may delay setting and early-age strength development, whereas excessive BFS replacement may accelerate hydration, producing high heat evolution that can induce thermal gradients, shrinkage, and microcracking. Furthermore, high FA contents may leave unreacted particles within the matrix and increase susceptibility to carbonation due to the depletion of the alkaline reserve [26,27,28,29,30]. The incorporation of NZ can partially mitigate these drawbacks because its porous structure provides an internal curing effect that helps reduce autogenous shrinkage and microcrack formation while improving moisture retention [11,13,31].

These complementary mechanisms suggest that combining multiple SCMs may improve mechanical performance while reducing embodied carbon of cementitious binders. However, identifying an optimal level combination within a predefined experimental design space remains a major challenge for multi-component binder systems, as the number of possible mixtures and interactions increases with each material. Traditional one-factor-at-a-time (OFAT) experimental methods are inefficient for such systems, requiring a prohibitive number of mix trials while failing to capture interactions among variables. Therefore, systematic design-of-experiments (DoE) techniques are required to efficiently screen multi-component SCM combinations.

Among the available DoE methods, the Taguchi orthogonal array method has been widely recognized as an efficient DoE approach capable of evaluating multiple variables with a reduced number of experimental trials. It has been extensively applied in geopolymer research to optimize parameters such as alkali activator composition, activator-to-binder ratio, alkali molarity, curing conditions, and precursor proportions [32,33,34,35,36]. However, its application to multi-component SCM replacement systems in cement mortars remains relatively limited.

To investigate the feasibility of multi-component cement mortars, this study employs a Taguchi L9 orthogonal array design incorporating natural zeolite (NZ), fly ash (FA), blast furnace slag (BFS), and calcium hydroxide (CH) as experimental factors. The flow value, compressive strength, density, and mechanical efficiency index (MEI) were evaluated to assess fresh-state properties and mechanical performance. Range analysis based on compressive strength results was conducted to determine the relative contribution of each factor and identify a favorable level combination within the investigated design space. Microstructural characterization was further performed to examine the mechanisms associated with different SCM combinations. Multiple linear regression analysis was applied to quantitatively describe the relationship between mixture parameters and compressive strength development and to compare predicted and experimental values. The identified level combination was experimentally validated, followed by an assessment of embodied carbon reduction based on OPC replacement ratios and an eco-efficiency index to evaluate the balance between mechanical performance and environmental impact.

## 2. Materials and Methods

### 2.1. Materials

**Figure 1:**
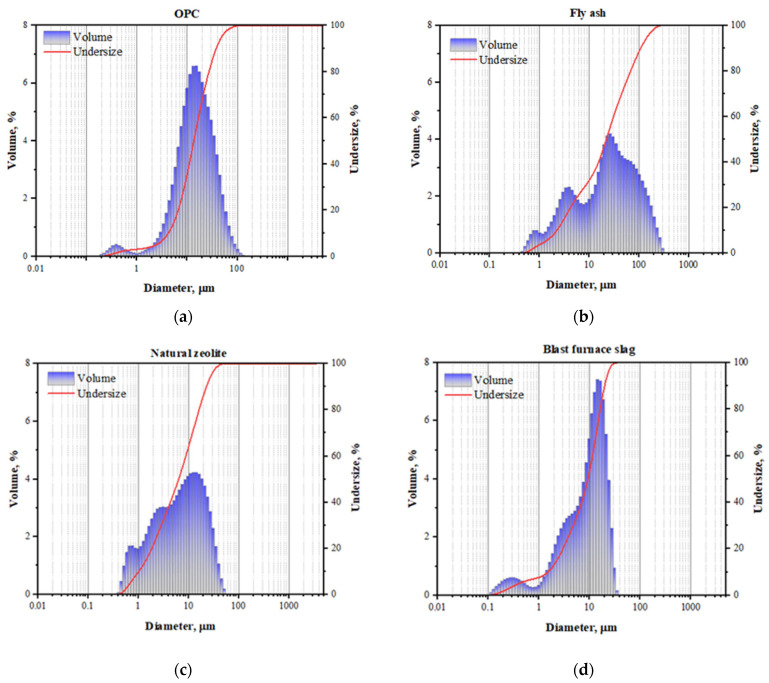
Particle size distributions of materials: (**a**) OPC, (**b**) fly ash, (**c**) natural zeolite, and (**d**) blast furnace slag.

This study used Ordinary Portland Cement (OPC), natural zeolite (NZ), fly ash (FA), blast furnace slag (BFS), and calcium hydroxide (CH) as the binder components. The chemical compositions of these materials, determined via X-ray fluorescence (XRF) analysis using a ZSX- Primus IV spectrometer (Rigaku, Tokyo, Japan), are presented in Table 1. Their characteristic particle size parameters are summarized in Table 2, and the particle size distributions measured using a laser particle size analyzer (Malvern Panalytical, Malvern, UK) are shown in Figure 1.

The OPC was supplied by Sungshin Co., Ltd. (Danyang, Republic of Korea), complied with KS L 5201 specifications, and exhibited a specific surface area of 3.45 m^2^/g and a specific gravity of 3.20. The natural zeolite, primarily composed of clinoptilolite, was obtained from Jinnong Industry (Pohang, Gyeongsangbuk-do, Republic of Korea). It exhibits a specific surface area of 4.30 m^2^/g and a density of 0.85 g/cm^3^, with a relatively fine and well-graded particle size distribution. The fly ash was sourced from the Boim Combined Heat and Power Plant (Yeosu, Republic of Korea), with a specific surface area of 3.87 m^2^/g and a density of 2.28 g/cm^3^. Its spherical particles exhibit a relatively broad particle size distribution compared to the other SCMs. The ground granulated blast furnace slag was supplied by Henan Dingnuo Purification Material Co., Ltd. (Gongyi City, Henan Province, China), exhibiting a specific surface area of 4.29 m^2^/g and a density of 2.86 g/cm^3^, with relatively fine and uniform particle distribution. Finally, extra-pure calcium hydroxide (98.2% purity), supplied by Daejung Chemical Co., Ltd. (Siheung, Republic of Korea), was incorporated at 2wt%–4 wt% of the OPC.

### 2.2. Mixture Design

The mixture designs for the multi-component cement mortars were developed using the Taguchi orthogonal array method. In this study, OPC was partially replaced by four supplementary cementitious materials, including NZ, FA, BFS, and CH. Four experimental factors (NZ, FA, BFS, and CH) were investigated at three levels each, and the selected factors and their levels are summarized in Table 3.

The Taguchi orthogonal array method provides an efficient approach for evaluating the effects of multiple variables while significantly reducing the number of experimental trials. Accordingly, an L9 (3^4^) orthogonal array was employed, requiring only nine experimental mixtures. The corresponding orthogonal array matrix is presented in Table 4, and the detailed mixture proportions are provided in Table 5.

All mortar specimens were prepared in accordance with ASTM C109 [37]. To ensure consistency among all mixtures, the binder-to-sand ratio was maintained at 1:2.75, and the water-to-binder (W/B) ratio was fixed at 0.485 throughout the study. The replacement levels of OPC by NZ, FA, BFS, and CH were defined based on the OPC mass, and the corresponding amounts of OPC and SCMs were adjusted to maintain a constant total binder mass.

### 2.3. Methods

#### 2.3.1. Physical and Mechanical Properties

The workability of the cement mortar was evaluated using a flow test in accordance with ASTM C1437-20 [38]. The compressive strength was measured using 50 mm cube specimens in accordance with ASTM C109 at curing ages of 3, 7, 14, and 28 days. Three specimens were tested for each mixture, and the average value was reported. The apparent density of the mortar specimens was measured at 28 days in accordance with ASTM C642 [39]. Based on the 28-day compressive strength and apparent density, a mechanical efficiency index (MEI) was calculated to evaluate the strength-to-weight performance of the mortar mixtures:

(1) $$MEI=\frac{fc}{\rho }$$

where fc is the 28-day compressive strength (MPa) and ρ is the apparent density (g/cm^3^). A higher MEI value indicates better mechanical efficiency.

#### 2.3.2. Factors Influence Analysis

Factor influence analysis was conducted to evaluate the effects of SCM factors (NZ, FA, BFS, and CH) on compressive strength development. Range analysis, main effect and combined trend analyses were performed to identify the relative influence and variation tendencies of individual and combined factors. Multiple linear regression (MLR) analysis was further applied to quantitatively describe the relationship between SCM factors and compressive strength. The regression model performance was evaluated using the coefficient of determination (R^2^) and relative error.

#### 2.3.3. Microstructure Analysis

Following the compressive strength tests, approximately 1 cm fragments were collected from the fractured specimens for microstructural characterization. The samples were coated with platinum (Pt) and examined using field-emission scanning electron microscopy (FE-SEM, SIGMA 360, Carl Zeiss, Oberkochen, Germany). For X-ray diffraction (XRD) analysis, additional specimen fragments were ground into powder to identify the hydration products. XRD measurements were performed using a D8 Advance diffractometer (Bruker AXS, Karlsruhe Germany).

#### 2.3.4. Environmental Assessment

In this study, the embodied carbon emissions of the multi-component cement mortar mixtures were evaluated based on the Global Warming Potential (GWP), expressed as kg CO_2_-eq. The GWP of each mixture was calculated using the following equation:

(2) $$GWP=\sum \left(M_{i}\times EF_{i}\right)$$

where $M_{i}$ is the mass proportion of the i-th component material within 1 kg of total binder, and $EF_{i}$ is the corresponding emission factor (kg CO_2_-eq/kg).

The emission factors of the constituent materials were obtained from previously reported literature and publicly available databases. The carbon emission factor of the OPC (Sungshin) was adopted as 0.769 kg CO_2_-eq/kg [40]. For natural zeolite, considering excavation, grading, milling, and on-site transportation processes, an average value of 0.05 kg CO_2_-eq/kg was used [41]. Industrial by-products, including fly ash and blast furnace slag, were assigned lower emission factors of 0.027 kg CO_2_-eq/kg [42] and 0.052 kg CO_2_-eq/kg [43], respectively. In contrast, calcium hydroxide powder exhibited a higher emission factor of 0.92 kg CO_2_-eq/kg due to its relatively energy-intensive production process [44].

To evaluate the balance between environmental impact and mechanical performance, an Eco-Efficiency Index (EEI) was introduced. This EEI was calculated using the following equation:

(3) $$\mathrm{EEI}\;=\;\frac{fc\;}{GWP\;}$$

where fc is the 28-day compressive strength (MPa), and GWP is expressed in kg CO_2_-eq binder. A higher EEI value indicates a more favorable balance between mechanical performance and environmental impact.

## 3. Results and Discussion

### 3.1. Workability

Table 6 presents the flow table test results of the multi-component cement mortars prepared using the L9 Taguchi orthogonal array. The flow values of all mixtures ranged from approximately 90.75% to 109.25%. The control mortar, containing 100% OPC, exhibited an average flow of approximately 105%. Several mixtures (M2, M3, M5, and M6) showed comparable or slightly higher flow values than the control mixture, with M3 achieving the maximum flow value of 109.25%. Conversely, mixtures M4 and M8 exhibited the lowest workability at 91.25% and 90.75%, respectively. The lower flowability observed in these mixtures can be associated with the combined effects of SCM type and replacement level. These mixtures typically incorporate medium-to-high NZ replacement levels with relatively lower FA and BFS contents. This trend is attributed to the distinct physical properties of the SCMs. While FA and BFS particles can contribute to improved flowability through their spherical morphology and particle effects, zeolite particles generally increase water demand due to their porous structure and higher water absorption capacity, which may increase internal friction within the mortar matrix [11,45,46]. Overall, most mixtures exhibited flow values close to the target range, suggesting that the selected W/B ratio of 0.485 was suitable for maintaining the required workability of the investigated mortar mixtures without additional adjustment.

### 3.2. Compressive Strength

**Figure 2:**
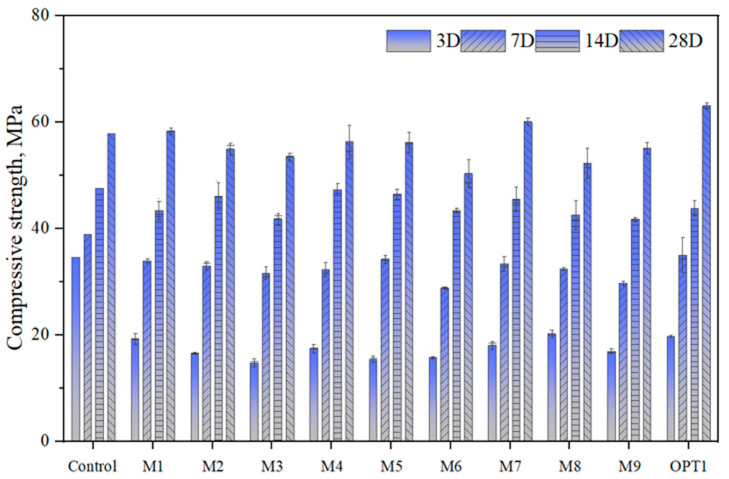
Compressive strengths of the multi-component cement mortars.

Figure 2 presents the compressive strengths of the multi-component cement mortars. At 3 days, the compressive strength ranged from 14.70 MPa to 20.17 MPa, representing a reduction of 48.2–62.2% compared with the control mixture (38.9 MPa). Mixture M1 exhibited the highest strengths, whereas M3 showed the lowest value. M1 contained the highest OPC content (73%), while M3 had the lowest (59%), indicating that early-age strength was primarily governed by OPC hydration. At 7 days, compressive strength ranged from 28.80 MPa to 34.20 MPa, corresponding to a reduction of 12.1–26.0% compared with the control mixture (38.9 MPa). The highest strengths were obtained for M1 and M5, while M6 exhibited the lowest value. Despite containing only 61% OPC, M5 achieved a compressive strength comparable to M1, indicating that the higher BFS content effectively compensated for the reduced cement content. In contrast, M6 contained a higher content of OPC (66%) but also higher FA content and lower BFS content, suggesting that BFS contributed more effectively than FA to early-age strength development due to its latent hydraulic reactivity. Although the L9 mixtures exhibited lower strength than the control mortar at 7 days, they maintained relatively high strength levels despite the substantial reduction in OPC content. At 14 days, compressive strength ranged from 41.67 MPa to 47.30 MPa. The highest strengths were observed in M4 (66% OPC) and M5 (61% OPC), although their OPC contents differed by approximately 5%. This result indicates that the influence of SCMs became increasingly significant as pozzolanic reactions progressed. Conversely, M3 (59% OPC) and M9 (62% OPC) exhibited the lowest strengths, despite having OPC contents comparable to M5. Their relatively higher FA contents likely delayed strength development because of the slower pozzolanic reactivity of fly ash. M4 mixture (66% OPC) exhibited a compressive strength of 47.3 MPa, which was only 0.42% lower than that of the control mixture (47.5 MPa), indicating that comparable 14-day strength could be achieved despite a substantial reduction in OPC content. At 28 days, compressive strength ranged from 50.30 MPa to 60.05 MPa, with M7 (61% OPC) exhibiting the highest value and M6 (66% OPC) the lowest. Although M7 contained only 61% OPC, it achieved the greatest compressive strength, which may be attributed to the combined contribution of higher NZ and BFS contents and the relatively lower FA content. These results indicate that the synergistic interaction between NZ and BFS effectively enhanced later-age strength through continued pozzolanic and hydraulic reactions. M7 mixture achieved a compressive strength of 60.05 MPa, representing a 3.84% increase compared with the control mixture (57.83 MPa), despite containing only 61% OPC.

Overall, the experimental results demonstrate a gradual shift in the dominant strength development mechanism with curing age. During the early curing period (3–7 days), compressive strength was primarily controlled by OPC hydration. As curing progressed to 14 days and 28 days, the continued pozzolanic reactions of NZ, together with the latent hydraulic reaction of BFS, promoted the formation of additional C-S-H gels, leading to progressive matrix densification and enhanced compressive strength [47,48]. Furthermore, the particle size distribution of the SCMs significantly influenced their reactivity and strength contribution. The relatively fine and uniformly distributed particles of NZ and BFS, which were comparable in size to OPC particles, may have facilitated improved particle packing and provided greater reaction potential within the cement matrix. In contrast, the broader particle size distribution and coarser particles of FA may have limited its early-age contribution to strength development due to its slower reaction rate. These physical characteristics, together with the distinct hydration mechanisms of each SCM, explain the differences in compressive strength observed among the L9 mixtures throughout the curing period.

### 3.3. Density

**Figure 3:**
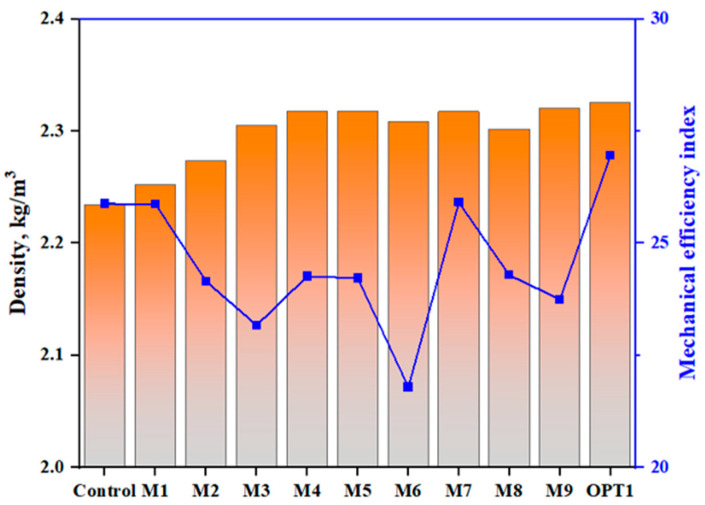
Density and mechanical efficiency index of the multi-component cement mortars.

Figure 3 presents the density and the 28-day compressive strength-to-density ratio of the control mortar and the Taguchi-designed mixtures (M1–M9). The density of all mixtures ranged from 2.23 g/cm^3^ to 2.32 g/cm^3^, indicating only minor variations despite the incorporation of different SCMs. Compared with the control mortar (2.23 g/cm^3^), all mixtures exhibited a slight increase in density, with the highest value of 2.32 g/cm^3^ observed in M4, M5, M7, and M9, corresponding to an increase of approximately 4.0%.

The 28-day compressive strength-to-density ratio was calculated to evaluate the mechanical efficiency of each mixture by relating its compressive strength to its density. The control mixture exhibited a ratio of 25.88, while M7 showed the highest value among the L9 mixtures (25.91), indicating the highest compressive strength relative to its density. In contrast, M6 exhibited the lowest ratio (21.79).

Overall, the density values of all mixtures were similar, whereas the compressive strength-to-density ratio showed greater variation among the mixtures. These results suggest that differences in the strength-to-density ratio were associated with the different SCM combinations used in the mixtures. The results also indicate that optimizing the SCM proportions can improve compressive strength while maintaining a similar mortar density.

### 3.4. Range Analysis

Range analysis was performed to evaluate the influence of each factor on the compressive strength of the multi-component cement mortar and to identify the optimal factor-level combination. The calculated *K* values, representing the average response at each factor level, together with the corresponding range values (R), are summarized in Table 7.

At 3 days, factor B (FA) exhibited the largest range value (R = 2.44). Factor C (BFS) and A (NZ) showed intermediate effects, whereas factor D (CH) had the smallest influence. Accordingly, the order of influence was B > C > A > D, and the optimal factor-level combination was A_3_B_1_C_1_D_3_. At 7 days, factor B remained the most influential factor (R = 3.17), followed by C, A, and D. The influence order remained B > C > A > D, while the optimal factor-level combination changed to A_1_B_2_C_3_D_1_.

At 14 days, factor B continued to exhibit the largest range value (R = 3.12). Factor A ranked second, followed by factors C and D, resulting in an influence order changed to B > A > C > D, and the corresponding optimal factor-level combination was A_2_B_1_C_2_D_2_. At 28 days, factor B again showed the highest range value (R = 5.25). The influence order remained B > A > C > D, and the optimal factor-level combination was A_3_B_1_C_3_D_1_.

Overall, factor B (FA) consistently exhibited the largest range values at all curing ages, indicating that it had the greatest effect on compressive strength within the investigated factor levels. Since 28-day compressive strength is generally regarded as the primary performance indicator for cement mortar, the optimal factor-level combination A_3_B_1_C_3_D_1_ was selected for subsequent validation and is hereafter referred to as OPT1.

### 3.5. Factor Influences Trends on Compressive Strength

**Figure 4:**
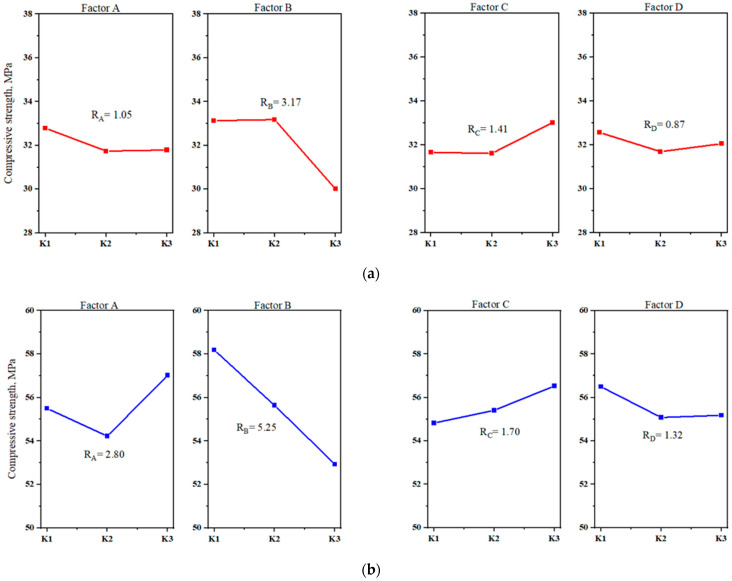
Factor response plots for compressive strength: (**a**) 7 days and (**b**) 28 days.

To further evaluate the factor-dependent trends in compressive strength, the results were categorized into two stages: early-age strength (3 days and 7 days) and later-age strength (14 days and 28 days). This classification is consistent with the range analysis, which showed different factors influence orders at different curing ages. The factor response plots for compressive strength at 7 days and 28 days are presented in Figure 4.

Factor A (NZ) exhibited a relatively limited effect on compressive strength at early ages. At 7 days, only minor variations in compressive strength were observed among the investigated replacement levels, suggesting a limited contribution of NZ during the early curing period. However, its effect became more apparent at 28 days, with the highest compressive strength observed at the 10% replacement level. This trend may be associated with the delayed pozzolanic reaction of NZ, which gradually consumes calcium hydroxide released during cement hydration and contributes to additional C–S–H formation during later curing stages [6,8,11].

Factor B (FA) showed the largest variation in compressive strength response among the investigated factors, as also indicated by the range analysis. Within the selected replacement levels, increasing FA content generally resulted in reduced compressive strength, particularly at 28 days. The lower strength observed at higher FA contents may be related to the relatively slower pozzolanic reaction of FA and the reduction in OPC content during the investigated curing period [49].

Factor C (BFS) exhibited a moderate positive trend with respect to compressive strength. At 7 days, the differences among the BFS replacement levels were relatively small, although a slight increase was observed at the 40% replacement level. At 28 days, increasing BFS content generally improved compressive strength. This behavior may be associated with the latent hydraulic characteristics of BFS, which gradually participate in hydration reactions under alkaline conditions and contribute to additional hydration products and matrix densification [50].

Factor D (CH) showed the smallest variation in compressive strength response at both early and later curing ages. A slight improvement was observed at the lowest replacement level (2%), whereas further increases in CH content resulted in limited changes. This indicates that increasing CH content beyond a certain level provided limited additional benefit within the investigated mixtures. One possible explanation is that externally added calcium hydroxide may not be fully available for subsequent pozzolanic reactions because part of the CH can dissolve into the curing water during immersion curing [51].

**Figure 5:**
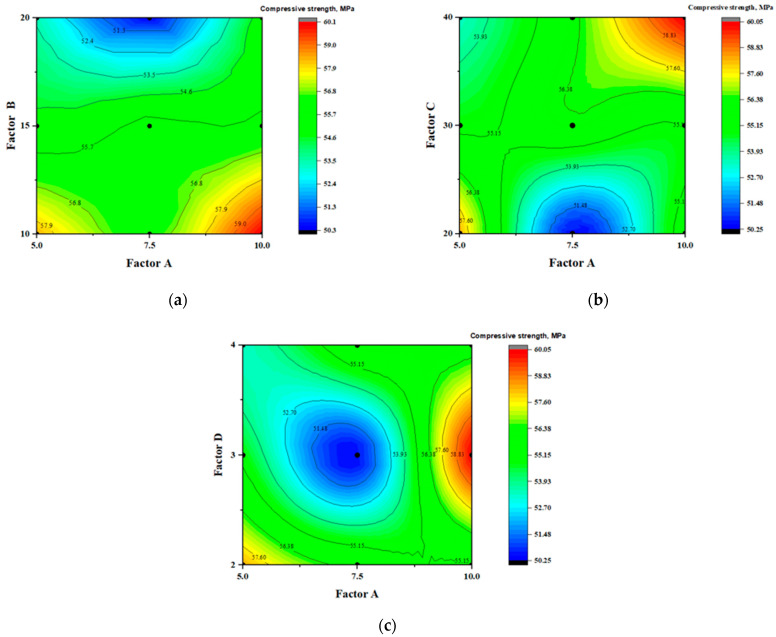
Contour plots showing the combined trends of two factors: (**a**) factor A and B, (**b**) factor A and C, and (**c**) factor A and D.

Figure 5 presents contour plots illustrating the apparent combined trends of two factors on the 28-day compressive strength. The contour plot of factors A and B indicates a nonlinear variation in compressive strength within the investigated factor levels. Higher strength regions, reaching approximately 58 MPa–60 MPa, were observed at the combination of low FA content (10%) and high NZ content (10%). In contrast, increasing FA content to 20% was associated with lower compressive strength across different NZ levels. This trend may be related to the slower reaction rate of FA and the reduction in OPC content at higher replacement levels during the investigated curing period.

The contour plot of factors A and C also shows an apparent combined trend. Higher compressive strength values were observed when higher levels of both NZ (10%) and BFS (40%) were incorporated, whereas combinations containing lower BFS levels exhibited relatively lower strength responses. This observation suggests that the simultaneous use of NZ and BFS may provide a favorable contribution within the investigated mixture combinations; however, further controlled experiments are required to verify their interaction effect.

Regarding the combined trend of factors A and D, the contour plot shows relatively smaller variations compared with other factor combinations. Although slightly higher compressive strength was observed at 10% NZ and 3% CH, the overall variation associated with CH remained limited within the investigated range. Overall, the contour plots illustrate the variation in compressive strength associated with different SCM combinations.

**Figure 6:**
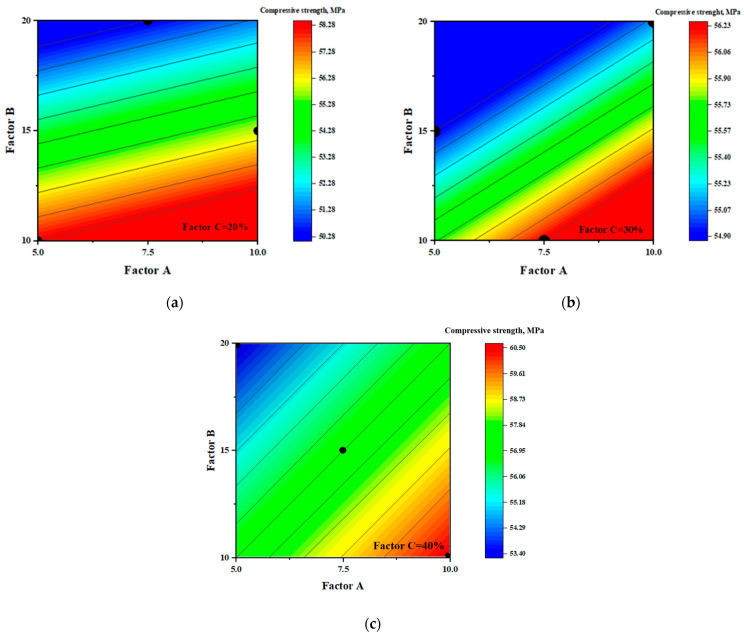
Contour plots showing the combined trend of three factors: (**a**) C = 20%, (**b**) C = 30% and (**c**) C = 40%.

Since range analysis indicated that factor D exhibited the smallest variation in compressive strength response, only factors A, B, and C were considered for further combined trend analysis. Figure 6 presents the contour plots illustrating the combined trends of these three factors. The contour plots show that factors A and B exhibited different response trends within the investigated design space. Increasing factor A was associated with higher compressive strength, whereas increasing factor B generally resulted in lower strength responses. At factor C = 20%, the reduction in compressive strength with increasing factor B was more apparent. As factor C increased to 30%, the contour distribution indicated a more favorable response region at higher levels of factor A and lower levels of factor B. At factor C = 40%, relatively higher compressive strength values (approximately 60 MPa) were observed. These observations suggest that the compressive strength response was affected by the combined variation in NZ, FA, and BFS within the investigated mixture combinations. In particular, higher BFS levels were associated with improved strength responses, while the contribution of NZ appeared more favorable under higher BFS conditions.

### 3.6. Microstructure Analysis

#### 3.6.1. SEM Analysis

**Figure 7:**
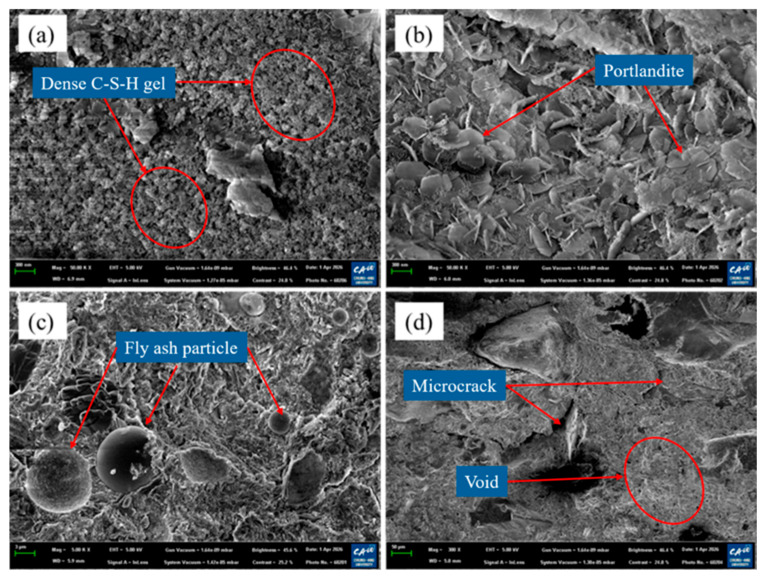
SEM pictures of the multi-component cement mortars: (**a**) M7 and (**b**), (**c**), (**d**) M6.

SEM analysis was performed on 28-day specimens of the multi-component cement mortars to evaluate the microstructural characteristics of the mixtures exhibiting the highest and lowest compressive strengths (M7 and M6), as shown in Figure 7. Figure 7a, obtained at a magnification corresponding to a 300 nm scale bar, shows that the M7 mixture possesses a dense and compact microstructure. A well-developed, interconnected C-S-H gel with a globular, sponge-like morphology forms a continuous matrix, while only a small amount of portlandite (CH) is observed. The limited CH content suggests that calcium hydroxide generated during cement hydration was consumed through the pozzolanic reactions of natural zeolite and fly ash, together with the latent hydraulic reaction of blast furnace slag, promoting the formation of additional secondary C-S-H gel. Consequently, the hydration products effectively filled the pore spaces and produced a refined and compact microstructure, which is consistent with the superior compressive strength of M7. In contrast, Figure 7b (300 nm scale bar) shows that the M6 mixture contains abundant, relatively large portlandite crystals and a noticeably less compact hydration matrix. The hydration products are discontinuously distributed, leaving visible voids between adjacent phases and indicating weaker internal bonding than that observed in M7. Figure 7c, acquired with a 3 μm scale bar, further reveals the presence of partially unreacted fly ash particles embedded within the cement matrix. These particles indicate that a portion of the fly ash remained insufficiently reacted at 28 days, reducing its contribution to strength development. The surrounding C-S-H gel also appears less continuous and less densely packed than that of M7. At a lower magnification (50 μm scale bar), Figure 7d shows numerous microvoids and microcracks distributed throughout the M6 matrix. A distinct interfacial transition zone (ITZ) between the aggregate and the surrounding cement paste is also observed, suggesting relatively weak interfacial bonding. These microstructural features, including the presence of unreacted fly ash particles, abundant CH crystals, microvoids, microcracks, and a weak ITZ, collectively account for the inferior compressive strength of M6. Overall, the SEM observations demonstrate that the formation of a dense, continuous C-S-H matrix accompanied by reduced portlandite content, fewer defects, and improved interfacial bonding contributes significantly to the enhanced mechanical performance of the optimized mortar mixture.

#### 3.6.2. XRD Analysis

**Figure 8:**
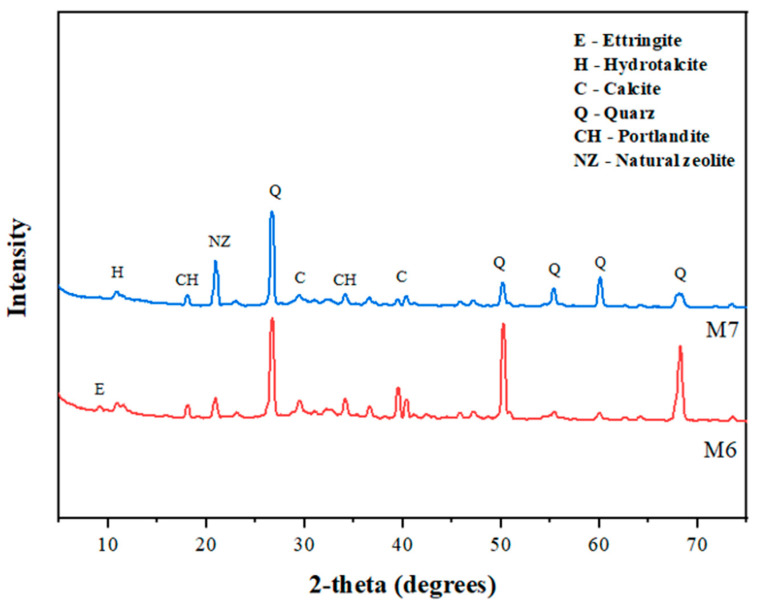
XRD patterns of the multi-component cement mortars.

XRD analysis was conducted on 28-day cured specimens of the multi-component cement mortars, focusing on the mixtures exhibiting the lowest and highest compressive strengths (M6 and M7), as presented in Figure 8. The XRD results provide further evidence to explain the relationship between phase evolution, microstructural development, and mechanical performance. For the M6 mixture, diffraction peaks corresponding to ettringite (AFt) are observed at approximately 9° (2θ), which agrees with the needle-like crystalline phases identified in the SEM images. In addition, relatively higher intensity peaks attributed to portlandite (CH) are detected at approximately 18° and 34° (2θ), indicating a considerable amount of residual calcium hydroxide within the hydrated matrix. The presence of remaining CH suggests that the pozzolanic reaction of natural zeolite and fly ash, as well as the latent hydraulic reaction of blast furnace slag, were comparatively limited in M6, resulting in insufficient formation of secondary C-S-H gel and a less compact microstructure.

In contrast, the M7 mixture exhibits substantially reduced CH peak intensity, indicating greater consumption of calcium hydroxide through the synergistic reactions among natural zeolite, fly ash, and blast furnace slag. The enhanced formation of secondary C-S-H gel is consistent with the dense and continuous C-S-H matrix observed in the SEM analysis, which contributed to the superior compressive strength of M7. Furthermore, due to the higher BFS content in M7, hydrotalcite-like phases are identified within the 10°–12° (2θ) range, which are associated with the incorporation of Mg^2+^ and Al^3+^ released during slag hydration. The formation of these secondary phases may further contribute to matrix refinement and improved stability of the hydrated structure. Calcite peaks are observed in both mixtures, mainly around 29° (2θ), which are attributed to carbonation of portlandite during sample preparation and exposure. However, the lower calcite intensity in M7 suggests that a larger proportion of CH participated in pozzolanic and slag hydration reactions rather than carbonation. Overall, the XRD results support the SEM observations and compressive strength results by demonstrating that optimized phase evolution, including reduced CH content and enhanced secondary hydration product formation, was responsible for the improved mechanical performance of the M7 mixture.

### 3.7. Statistical Analysis

A multiple linear regression analysis was performed using IBM SPSS Statistics version 28.0 to quantitatively evaluate the relationship between SCM content and compressive strength development at different curing ages. Table 8 presents the regression coefficients and corresponding R^2^ values at different curing ages. The regression coefficients indicate that the effects of SCM components varied with curing age. At 3 days, factors A (+0.309) and D (+0.138) showed positive contributions, whereas factors B (−0.244) and C (−0.117) exhibited negative contributions. These results suggest that NZ and CH may have contributed to early-age strength development, while FA and BFS showed limited early-age contribution due to their slower reaction characteristics. At 7 days, most factors exhibited negative coefficients, with the largest reductions observed for factors B (−0.312) and D (−0.255). This suggests that higher replacement levels of these components may decrease early-age strength within the investigated range. At 14 days, the regression coefficients became relatively small, and the lower R^2^ value indicates a transitional stage where the individual effects of SCM components became less distinguishable due to the combined effects of cement hydration, pozzolanic reactions, and slag hydration. At 28 days, factor A (+0.299) and factor C (+0.085) showed positive contributions, whereas factors B (−0.525) and D (−0.662) exhibited negative contributions. These results suggest that excessive FA and CH contents may negatively affect long-term strength development within the investigated range. The higher R^2^ value at 28 days indicates improved model consistency, reflecting the more stable contribution of OPC hydration, NZ-related pozzolanic reactions, and BFS hydration reactions at later ages.

## 4. Performance Evaluation and Environmental Impact

### 4.1. Optimized Mixture (OPT1)

Based on the range analysis of compressive strength derived from the L9 orthogonal array, the optimized mixture was identified as A_3_B_1_C_3_D_1_, corresponding to 10% NZ, 10% FA, 40% BFS, and 2% CH. The compressive strength of this optimized mixture (OPT1) is presented in Figure 3. The OPT1 exhibited higher compressive strength than all nine initial mixtures, particularly at 7 days and 28 days, reaching 34.97 MPa and 62.67 MPa, respectively. Furthermore, the 28-day compressive strength of OPT1 was 8.4% higher than that of the control mortar prepared with 100% OPC.

### 4.2. Regression Analysis

**Figure 9:**
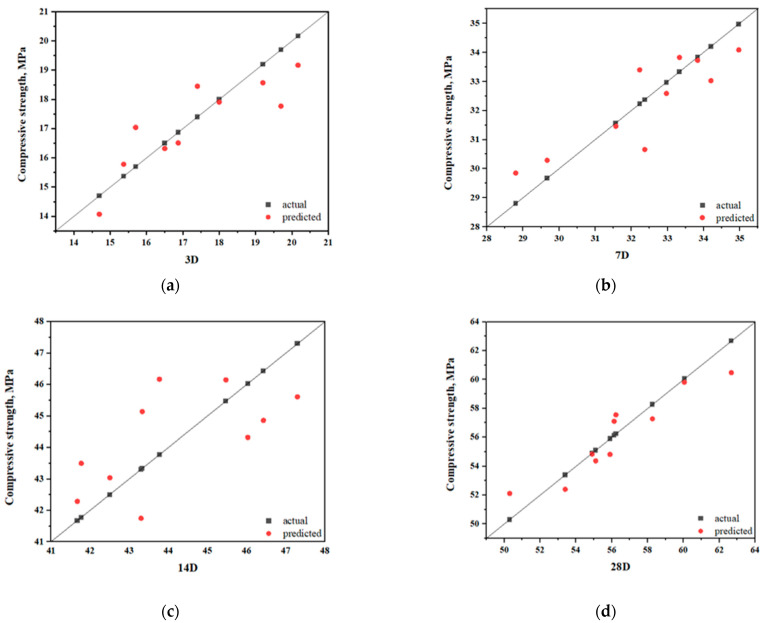
Comparative analysis of actual and predicted values. (**a**) 3 days, (**b**) 7 days, (**c**) 14 days, and (**d**) 28 days.

Based on the multiple linear regression analysis, regression equations were established to describe the relationship between SCM factors and compressive strength development at 3, 7, 14, and 28 days. The calculated values from the regression equations were compared with the experimental results of the L9 mixtures and the optimized mixture (OPT1), as presented in Table 9. The correlation between experimental and calculated values is illustrated in Figure 9.

The regression results showed good agreement between the calculated and experimental compressive strength values, with R^2^ values ranging from 0.507 to 0.842. The highest R^2^ value was obtained at 28 days, suggesting that the regression model effectively captured the relationship between SCM composition and long-term compressive strength development.

The relative differences between calculated and experimental values were within 0.47–8.55%, 0.30–5.29%, 1.28–4.19%, and 0.11–3.59% at 3 days, 7 days, 14 days, and 28 days, respectively. These results confirm that the regression equations can effectively represent the relationship between SCM composition and compressive strength development within the investigated experimental range.

### 4.3. Environmental Impact

**Figure 10:**
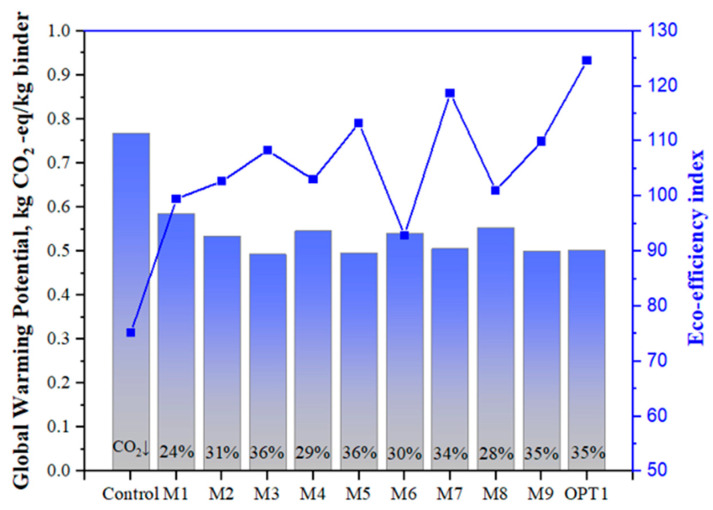
Global Warming Potential and Eco-efficiency index.

In this section, the environmental impact of the multi-component binder system was evaluated based on the Global Warming Potential (GWP), expressed as kg CO_2_-eq/kg binder, and the corresponding eco-efficiency index (EEI), as illustrated in Figure 10. A functional unit of 1 kg of binder was defined for the environmental assessment to ensure a consistent comparison among all mixtures.

The GWP of the control mixture (100% OPC) was calculated as 0.769 kg CO_2_-eq/kg binder. In comparison, all mixtures designed using the L9 orthogonal array exhibited substantially lower GWP values. The lowest GWP values were obtained for mixtures M3 and M5, with values of 0.493 and 0.495 kg CO_2_-eq/kg binder, respectively, representing reductions of approximately 36% compared with the control mixture. Among the L9 mixtures, M1 exhibited the highest GWP value (0.586 kg CO_2_-eq/kg binder); however, this value still corresponds to a 24% reduction compared with the OPC reference. The optimized mixture (OPT1) also demonstrated a reduced GWP value of 0.503 kg CO_2_-eq/kg binder, corresponding to an approximately 35% reduction compared with the control mixture.

In addition to the absolute GWP values, the eco-efficiency index (EEI) was evaluated to assess the relationship between environmental impact and mechanical performance. Among the L9 mixtures, M7 achieved the highest EEI value of 118.7, indicating the most favorable combination of compressive strength performance and reduced environmental impact. Mixtures M3 and M5 also exhibited relatively high EEI values, demonstrating their potential as low-carbon cementitious materials. Furthermore, OPT1 achieved the highest EEI value of 124.6, indicating an improved balance between mechanical performance and environmental impact. These results suggest that the optimized multi-component binder system can reduce environmental impact while maintaining competitive mechanical performance, demonstrating its potential for sustainable construction applications.

## 5. Conclusions

This study experimentally optimized the mixture proportions of multi-component cement mortars incorporating natural zeolite (NZ), fly ash (FA), blast furnace slag (BFS), and calcium hydroxide (CH). The Taguchi orthogonal array method and range analysis were employed to determine the optimal mixture proportions, and the optimized mixture was further evaluated through microstructural characterization, mechanical efficiency analysis, and environmental assessment. In addition, multiple linear regression analysis was conducted to quantitatively evaluate the influence of SCM factors on compressive strength development at different curing ages. Based on the experimental results, the following conclusions can be drawn:

1. The optimized mixture (OPT1), consisting of 10% NZ, 10% FA, 40% BFS, and 2% CH, exhibited the highest mechanical performance among the investigated mixtures. At 28 days, its compressive strength exceeded those of all L9 experimental mixtures and 100% OPC control mortar, showing an 8.4% improvement compared with the control mixture.
2. Multiple linear regression analysis quantitatively described the relationship between SCM factors and compressive strength development, with R^2^ values ranging from 0.507 to 0.842. The variation in regression performance among curing ages indicates the influence of different hydration mechanisms, including the transition from OPC hydration-dominated strength development at early ages to more complex hydration and pozzolanic reactions at later ages.
3. The optimized mixture (OPT1) reduced the Global Warming Potential (GWP) by approximately 35% compared with the control mixture. Furthermore, OPT1 achieved the highest eco-efficiency index (EEI = 124.6), which was 1.6 times higher than that of the control mixture, indicating an improved balance between mechanical performance and environmental impact.

Overall, the findings indicate that the Taguchi-based mixture optimization approach is an effective method for designing high-performance, low-carbon cement mortars. The optimized multi-component cement mortar system demonstrates potential for reducing the environmental impact of cement-based materials while maintaining competitive mechanical performance.

### Limitations and Future Work

This study primarily focused on compressive strength, microstructural development, and carbon reduction potential, as these parameters directly correspond to the objective of developing a low-carbon multi-component cementitious system. However, other important long-term performance aspects, particularly durability-related properties such as drying shrinkage, permeability, and resistance to chemical attack, were not investigated. Therefore, future research should evaluate these properties through long-term durability tests to further verify the performance and practical applicability of the proposed system.

## Figures and Tables

**Table 1:** Chemical compositions of materials.

Materials	Content (%)							
	CaO	SiO_2_	Al_2_O_3_	MgO	Fe_2_O_3_	Na_2_O	K_2_O	TiO_2_
OPC	63.40	19.70	4.90	2.60	3.70	0.20	1.00	0.30
Natural zeolite	2.32	66.70	15.20	1.80	3.03	1.93	1.98	0.34
Fly ash	3.00	54.8	30.50	1.10	4.60	0.90	1.80	2.0
Blast furnace slag	44.20	27.49	14.31	9.77	0.31	0.46	0.41	1.19

**Table 2:** Particle size parameters of materials.

Materials	d_10_ (μm)	d_50_ (μm)	d_90_ (μm)	Grading Width	Span
OPC	4.25	13.65	38.02	33.77	2.47
Natural zeolite	1.17	7.25	26.00	24.83	3.42
Fly ash	2.23	22.26	109.69	107.46	4.83
Blast furnace slag	1.52	9.36	20.03	18.51	0.31

**Table 3:** Level table of factors.

Level	Factor			
	NZ (A)	FA (B)	BFS (C)	CH (D)
1	5%	10%	20%	2%
2	7.5%	15%	30%	3%
3	10%	20%	40%	4%

**Table 4:** Taguchi L9 orthogonal array design.

Mix ID	Factor			
	A	B	C	D
M1	5%	10%	20%	2%
M2	5%	15%	30%	3%
M3	5%	20%	40%	4%
M4	7.5%	10%	30%	4%
M5	7.5%	15%	40%	2%
M6	7.5%	20%	20%	3%
M7	10%	10%	40%	3%
M8	10%	15%	20%	4%
M9	10%	20%	30%	2%
OPT1	10%	10%	40%	2%

**Table 5:** Mixture proportions of multi-component cement mortars.

Mix ID	Binder					Total Binder	Sand Aggregate	Water	Total Weight	OPC/Binder
	OPC	A	B	C	D					
Unit	(g)	(g)	(g)	(g)	(g)	(g)	(g)	(g)	(g)	-
Control	1000	0	0	0	0	1000	2750	485	4235	1.00
M1	739.9	36.5	73.0	146.0	14.6	1000	2750	485	4235	0.73
M2	653.6	32.7	95.0	196.1	19.6	1000	2750	485	4235	0.65
M3	591.7	29.6	118.3	236.7	23.7	1000	2750	485	4235	0.59
M4	660.1	49.5	66.0	198.0	26.4	1000	2750	485	4235	0.66
M5	607.9	45.6	91.2	243.2	12.2	1000	2750	485	4235	0.61
M6	664.5	49.8	132.9	132.9	19.9	1000	2750	485	4235	0.66
M7	613.5	61.4	61.4	245.4	18.4	1000	2750	485	4235	0.61
M8	671.1	67.1	100.7	134.2	26.9	1000	2750	485	4235	0.67
M9	617.3	61.7	123.5	185.2	12.3	1000	2750	485	4235	0.62
OPT1	617.3	61.7	61.7	246.9	12.4	1000	2750	485	4235	0.61

Binder: Sand = 1:2.75, Water/Binder = 0.485.

**Table 6:** Flow value of the multi-component cement mortars.

MIX ID	Measured Value, mm	Mean, mm	Flow, %
Control	203, 205, 205, 207	205	105.0
M1	202, 199.5, 199, 198	199.6	99.6
M2	207, 206, 205.5, 205	205.8	105.8
M3	210, 212, 207, 208	209.2	109.2
M4	189, 192, 192.5, 193	191.6	91.6
M5	211, 208, 206, 200	206.2	106.2
M6	206, 207, 207, 200	206.5	106.5
M7	203, 204, 202, 202.5	202.8	102.8
M8	187, 190, 192, 194	190.7	90.7
M9	194, 195, 199, 203	197.7	97.7
OPT1	196, 197, 198, 199	197.5	97.5

**Table 7:** Range analysis results.

Curing Ages	Factor	K_1_	K_2_	K_3_	R	Influence Order	Optimal Combination
3d Compressive strength	A	16.80	16.16	**18.35**	2.19	B > C > A > D	A_3_B_1_C_1_D_3_
	B	**18.20**	17.35	15.76	2.44		
	C	**18.36**	16.92	16.02	2.33		
	D	17.15	16.73	**17.42**	0.69		
7d Compressive strength	A	**32.79**	31.74	31.79	1.05	B > C > A > D	A_1_B_2_C_3_D_1_
	B	33.13	**33.18**	30.01	3.17		
	C	31.67	31.62	**33.03**	1.41		
	D	**32.57**	31.70	32.06	0.87		
14d Compressive strength	A	43.71	**45.68**	43.21	2.46	B > A > C > D	A_2_B_1_C_2_D_2_
	B	**45.37**	44.99	42.25	3.12		
	C	43.04	**45.00**	44.56	1.96		
	D	43.81	**44.93**	43.86	1.12		
28d Compressive strength	A	55.50	54.22	**57.02**	2.80	B > A > C > D	**A_3_B_1_C_3_D_1_** (OPT1)
	B	**58.18**	55.64	52.93	5.25		
	C	54.82	55.41	**56.53**	1.70		
	D	**56.50**	55.08	55.18	1.32		

**Table 8:** Regression coefficients and R^2^ values at different curing ages.

Age	Constant	A	B	C	D	R	R^2^
3 Day	21.53	+0.309	−0.244	−0.117	+0.138	0.90	0.805
7 Day	36.99	−0.200	−0.312	+0.068	−0.255	0.85	0.718
14 Day	47.29	−0.099	−0.312	+0.076	−0.023	0.71	0.507
28 Day	60.65	+0.299	−0.525	+0.085	−0.662	0.92	0.842

**Table 9:** Measured values vs. predicted values.

Mix ID	3d Compressive Strength, MPa			7d Compressive Strength, MPa			14d Compressive Strength, MPa			28d Compressive Strength, MPa		
	Measured	Predicted	Error/%	Measured	Predicted	Error/%	Measured	Predicted	Error/%	Measured	Predicted	Error/%
M1	19.20	18.57	3.27	33.83	33.73	0.30	43.33	45.14	4.19	58.27	57.27	1.71
M2	16.50	16.32	1.09	32.97	32.59	1.14	46.03	44.32	3.71	54.90	54.84	0.11
M3	14.70	14.07	4.30	31.57	31.46	0.35	41.77	43.50	4.14	53.40	52.40	1.87
M4	17.40	18.45	6.04	32.23	33.40	3.62	47.30	45.61	3.57	56.23	57.55	2.34
M5	15.37	15.78	2.70	34.20	33.03	3.43	46.43	44.86	3.39	56.13	57.10	1.73
M6	15.70	17.04	8.55	28.80	29.85	3.66	43.30	41.75	3.57	50.30	52.11	3.59
M7	18.00	17.91	0.47	33.33	33.83	1.51	45.47	46.15	1.49	60.05	59.81	0.40
M8	20.17	19.17	4.94	32.37	30.66	5.29	42.50	43.04	1.28	55.90	54.82	1.93
M9	16.87	16.51	2.15	29.67	30.29	2.08	41.67	42.29	1.49	55.10	54.37	1.32
OPT1	19.70	17.77	1.93	34.97	34.09	0.88	43.77	46.17	2.40	62.67	60.47	2.20

## Data Availability

The original contributions presented in this study are included in the article. Further inquiries can be directed to the corresponding author.
